# Network Pharmacological Study and Molecular Docking Analysis of Qiweitangping in Treating Diabetic Coronary Heart Disease

**DOI:** 10.1155/2021/9925556

**Published:** 2021-07-27

**Authors:** Tianfu Liu, Jianbo Liu, Liang Hao

**Affiliations:** ^1^China Medical University-The Queen's University of Belfast Joint Collage, China Medical University, Shenyang 110122, Liaoning Province, China; ^2^Shanxi Three-Principles & Six-Disease Institute of Traditional Chinese Medicine, Taiyuan 030001, Shanxi Province, China; ^3^Department of Chemistry, China Medical University, Shenyang 110122, Liaoning Province, China

## Abstract

**Background:**

Coronary heart disease (CHD) is one of the most important complications of diabetes mellitus, having a high disability fatality rate. Qiweitangping is a Chinese medicine to control diabetes (type 1 and type 2 diabetes) and complications, having been used in clinic for more than 20 years, with the expected therapeutic effect. In a previous clinical study, the total effective rate of the drug for the treatment of type 2 diabetes reached 92.7%. However, the mechanism of the treatment process is unclear. Therefore, this research was conducted to explore the mechanism of treating diabetic coronary heart disease with the assistance of bioinformatics methods.

**Methods:**

The TCMSP database was used to collect the effective chemical constituents of Qiweitangping and the target genes of the chemical constituents, and the related genes of diabetic CHD were obtained from the GeneCard database. Furthermore, the intersection was found between the target gene of the drug and the related gene of the disease to obtain the candidate genes; the STRING database and DAVID database were used to perform protein interaction analysis and KEGG enrichment analysis on the candidate genes. Also, molecular docking was used for auxiliary verification. Finally, a “drug component-gene target-pathway” network was constructed by using Cytoscape software.

**Results:**

Sixty-two effective chemical components including naringin, diosgenin, formogenin, isorolin, and isocryptanshinone, fifty-nine candidate target genes (such as AKT1, CASP3, and VEGF-A), and thirty-nine related pathways in Qiweitangping were obtained. In addition, two pairs (CASP-naringenin and STAT3-cryptotanshinone) of molecular docking results showed good affinity (<−5.00 kcal/mol).

**Conclusion:**

The results of the study indicate that Qiweitangping treats diabetic CHD with multiple chemical components. Its mechanism of action may be related to the HIF-1 signaling pathway, PI3K-Akt signaling pathway, and ErbB signaling pathway. STAT3 and CASP genes have been verified by molecular docking to have a relatively good combination with Qiweitangping. This study is the theoretical basis for further experimental study on the treatment mechanism of diabetic CHD with Qiweitangping.

## 1. Introduction

Diabetes is a metabolic disease characterized by high blood sugar. According to data released by the International Diabetes Federation in 2019, 463 million adults aged 20 to 79 have diabetes worldwide [1]. There are four main types of diabetes, of which type 1 diabetes and type 2 diabetes account for most diabetic patients. Type 1 diabetes was once called juvenile diabetes or insulin-dependent diabetes. The pancreas of patients with type 1 diabetes produces almost no insulin by itself. Type 2 diabetes is more common and occurs when the body becomes resistant to insulin or cannot produce enough insulin. The current medications for diabetes are mainly insulin therapy and oral hypoglycemic drugs. Type 1 diabetes requires insulin treatment. Type 2 diabetes patients are mainly treated with sulfonylureas, biguanides, alpha glucosidase inhibitors, insulin sensitizers, and glinide insulin secretagogues.

Diabetes can cause complications that cause severe damage to the heart, blood vessels, eyes, kidneys, and nerves, especially diabetic coronary heart disease (CHD). Diabetic CHD is one of the most common complications of diabetes [2]. In the diabetic population, 80% of patients die of cardiovascular disease, of which 2/3 die of coronary heart disease [3, 4]. A report issued by the Centers for Disease Control and Prevention in the United States in 2014 showed that, in the United States, the death rate of adult patients with cardiovascular disease is 1.7 times that of nondiabetic patients. Further research has concluded that the main cause of this situation is the higher risk of stroke and obstruction [5]. Therefore, it is necessary to study how to effectively prevent and treat diabetic CHD.

Liu Shaowu, a Chinese medicine practitioner, proposed a unique theory of traditional Chinese medicine (TCM). Liu's medical theory has been successfully used in his clinical practice. Qiweitangping is a Chinese medicine developed based on Liu's medical theory. Qiweitangping, made from 27 kinds of Chinese herbal medicine, is a medicine to control diabetes (type 1 diabetes and type 2 diabetes) and the complications. A clinical study of 150 diabetic patients [6] showed that Qiweitangping not only effectively improved the clinical symptoms of patients with type 1 diabetes or type 2 diabetes but also reduced the patients' dependence on other hypoglycemic drugs. In recent years, clinicians have discovered that Qiweitangping has a therapeutic effect on diabetic CHD. Therefore, it becomes meaningful to explore the mechanism of action of Qiweitangping in the treatment of diabetic CHD.

However, Qiweitangping's chemical components are relatively complex, for it is made from a variety of Chinese herbal medicine, which make the mechanism of treating disease difficult to study. Nowadays, with the assistance of bioinformatics methods, it is possible to study the mechanism of Qiweitangping's disease treatment.

**Figure 1:**
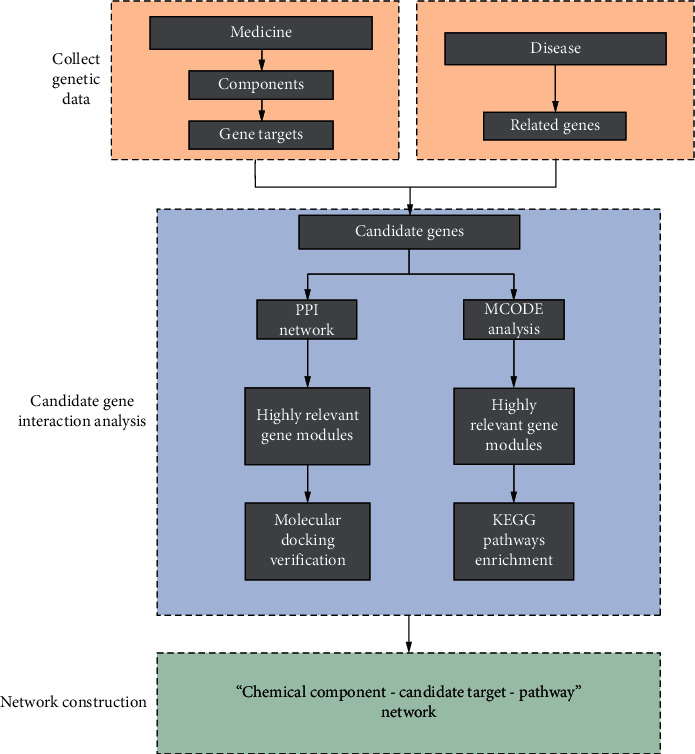
A flow chart to visualize the process.

This research used the network pharmacology method in bioinformatics to find potential mechanisms of Qiweitangping on the treatment of diabetic CHD and use molecular docking for auxiliary verification. Network pharmacology is an emerging discipline that reveals the effect of Chinese medicine on the body's regulatory network. Due to its holistic and systematic research methods and its emphasis on drug interactions, it is consistent with the characteristics of multitarget and multipathway in Chinese medicine. It has become an effective tool to systematically analyze the multitarget and multipath mechanism of traditional Chinese medicine compounds. The technical flow chart of this study is shown in Figure 1.

## 2. Materials and Methods

### 2.1. Collection and Screening of Effective Chemical Components of Qiweitangping

In the pharmacology database and analysis platform TCMSP (https://tcmspw.com/index.php) of traditional Chinese medicine system, twenty-seven Chinese herbal medicines were used as keywords to search and filter chemical composition. The database contains about 500 drugs included in the Pharmacopoeia of the People's Republic of China, which provides component absorption, distribution, metabolism, and excretion (ADME) data and target and disease information. In addition, the components not included in the TCMSP database were collected by querying the BATMAN (http://bionet.ncpsb.org/batman-tcm/) database. With reference to the relevant literature [7], this article used the basic screening principle defined as follows: oral bioavailability (OB) ≥ 30% and drug-likeness (DL) ≥ 0.18. To obtain the core active components, the filter conditions of OB increased to be greater than or equal to 50%. Microsoft Excel was used to perform comparison and deweighting analysis to construct a database of effective chemical components of Qiweitangping.

### 2.2. Construction of an Effective Chemical Component Target Gene Set and Diabetic-CHD-Related Gene Set

The gene targets corresponding to the effective chemical components were searched and exported through the TCMSP database to form a dataset of drug target genes. Target genes related to diabetic CHD were collected through the GeneCards (http://www.genecards.org/) database to form a disease gene dataset.

### 2.3. Screening of Candidate Targets for Qiweitangping in the Treatment of Diabetic CHD

Intersection genes, the common intersection data between the drug target gene dataset and the disease gene data set, were used as candidate target genes of Qiweitangping for the treatment of diabetic CHD.

### 2.4. PPI Analysis and Molecular Docking to Verify Binding Energy

Biomolecular functional annotation system STRING (https://string-db.org/) was used to construct a PPI (protein-protein interaction) network map of the proteins translated by the candidate target genes, and the connectivity value of each candidate target genes were calculated.

Three-dimensional structures of the target protein were downloaded from the PCSB PDB (http://www.rcsb.org) website, and the structural formula of the drug molecule was obtained from the TCMSP (https://tcmspw.com/tcmsp.php) and TCMID websites. AutoDockTools 4.2.6 software was used to process and dock the target protein and drug (http://119.3.41.228:8000/tcmid/) molecule, and PyMOL 2.7 software was used to visualize the results.

### 2.5. MCODE Analysis and Enrichment Analysis of Gene Clusters

MCODE function of Cytoscape 3.8.0 was used to analyze the candidate genes obtained by PPI analysis, and gene clusters with higher correlation were obtained.

Then, gene clusters were entered in the DAVID (https://david.ncifcrf.gov/) database, and KEGG enrichment analysis was performed.

### 2.6. Network Construction and Analysis

Regarding the candidate targets of Qiweitangping for the treatment of diabetic CHD and the effective chemical components of Qiweitangping obtained above, Cytoscape software was used to construct a “drug component-target-pathway-disease” network to realize Qiweitangping treatment visual research and analysis of the multicomponent and multitarget mechanism of diabetic CHD.

## 3. Results and Discussion

### 3.1. Component-Target Gene Network Analysis of Qiweitangping

**Figure 2:**
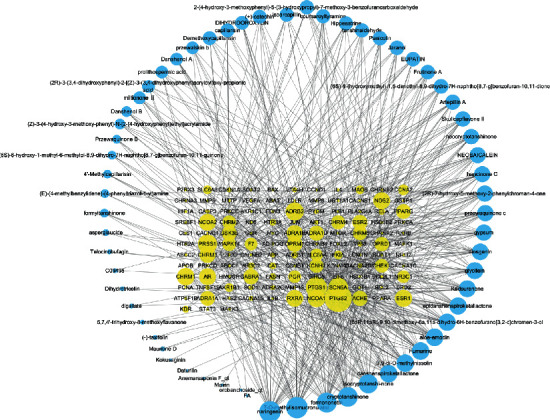
The composition and target network of Qiweitangping. The blue and yellow circles correspond to the chemical compositions and gene targets. The lines represent the relationship between the chemical composition and the target gene. The size of a node is proportional to the number of connected nodes.

In the network corresponding to the chemical components and target genes of Qiweitangping (Figure 2), there are a total of 186 nodes and 624 edges. Among them, 62 blue nodes represent chemical components and 124 yellow nodes correspond to targets. The specific information of 62 chemical components is provided in Table 1.

This suggests that Qiweitangping's mechanism of treating diseases may be related to these gene targets.

A study induced the formation of an endothelial dysfunction model through fructose feeding and used naringenin combined with neuroprotein (100 mg/(kg·d)) to intervene in animals for 4 weeks. The results show that naringenin can significantly increase the level of nitrate/nitrite (nitrogen oxide) and upregulate the expression of phosphorylated eNOS (p-eNOS) and endothelial nitric oxide synthase (eNOS), while the high fructose diet will reduce the expression of eNOS (p-eNOS) and endothelial nitric oxide synthase (eNOS) [8]. Naringenin provides protection against injury caused by hyperglycemia through antiapoptosis, antioxidation, antimitochondrial, antifibrosis, and anti-inflammatory effects [9]. Many risk factors and pathological processes are common in patients with endothelial dysfunction, including insulin resistance, atherosclerosis, dyslipidemia, hyperglycemia, and coronary artery disease. Naringenin improves endothelial function by reducing the accumulation of ROS in endothelial cells and increasing the production of NO [10].

Diosgenin has potential effects on the initiation and cell regeneration of *β* events caused by STZ [11]. Dioscin has a therapeutic effect on lipid deposition in the heart of diabetic rats and has a protective effect on the histological pathology of myocardial tissue in diabetes [12].

Isorhamnetin inhibits the instantaneous release of calcium ions in cells, thereby resisting the damage of oxidative damage to endothelial cells [13, 14].

Experimental studies have shown that the function of formononetin to relax blood vessels may be related to processes such as inhibition of voltage-dependent calcium channels, intracellular calcium ion release, activation of potassium ion channels, and NO release [15, 16].

### 3.2. Network Analysis of Candidate Genes

In the GeneCard database, 940 diabetic-CHD-related genes are collected. Then, 59 common genes (Table 2), obtained amongst 940 diabetic CHD genes and 124 drug-gene targets, were used as candidate target genes.

**Figure 3:**
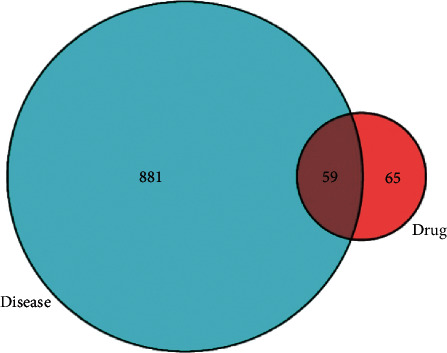
Schematic diagram of finding common genes.

In Figure 3, the blue circle on the left represents the related genes of diabetic CHD and the red circle on the right represents the gene target of Qiweitangping. The middle area represents the 59 common genes of drug gene targets and disease genes, corresponding to the core genes in the treatment.

**Figure 4:**
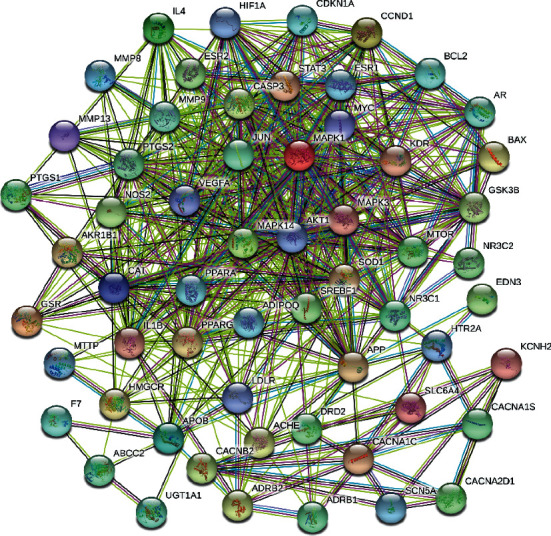
PPI network diagram.

We input the common gene information into the string database and get the protein-protein interaction (PPI) network diagram of these 59 genes (Figure 4). The statistical results of the data in the PPI network diagram (Table 3) show that AKT1, MAPK3, CASP3, etc. are important genes in this network.

Each circular node in Figure 4 represents a protein. The lines between nodes represent the interactions between nodes. The more interactions mean the protein is more important in the network.

The ordinate in Table 3 is the gene name. The abscissa is the degree of the gene, referring to the number of other nodes directly connected to a node in the network. The higher the degree is, the more important the node is. We only selected the genes with top 10 degrees to make this table.

From the analysis of the PPI network diagram (Table 3), the relationship between the candidate target genes of the drug Qiweitangping in the treatment of diabetic CHD is very complicated. The more relevant targets in the statistical network include AKT1, CASP3, and VEGF-A. These targets are mainly involved in the processes of apoptosis, angiogenesis, and inflammation.

AKT1 regulates the basic functions of vascular cell proliferation, differentiation, migration, and apoptosis and is very important for endothelial cell function and vascular integrity. Its mechanism may be related to the activation of the PI3K-Akt signaling pathway and upregulation of VEGF expression. It promotes angiogenesis and reduces the risk of myocardial ischemia caused by CHD [17].

CASP3 belongs to the subfamily of apoptotic proteases. Its activation mode is mainly through the damaged cell mitochondrial membrane, released from the cell to the outside of the cell to induce cell apoptosis [18].

VEGF-A specifically acts on vascular endothelial cells, promoting the proliferation and migration of vascular endothelial cells, inhibiting endothelial cell apoptosis, promoting angiogenesis and maintaining blood vessels, improving insulin resistance, etc. [19].

Studies have pointed out that silencing MAPK1 promotes autophagy and inhibits the further development of CHD [20].

STAT3 is an important part of the JAK/STAT3 signaling pathway. At the same time, STAT3 can have a beneficial effect on cardiomyocyte function by activating the mitochondria and transcriptional response, and it can protect the heart by regulating the cardiac microenvironment and the activity of cardiac fibroblasts, which may involve reducing oxygen consumption of cardiomyocytes, inhibiting cardiomyocyte apoptosis, promoting myocardial differentiation, anti-inflammatory, etc. [21, 23].

We further applied molecular docking in our research to verify the binding energy between the gene targets and drug compounds. The results of molecular docking are shown in Table 4.

**Figure 5:**
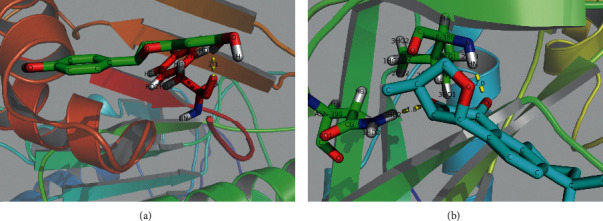
(a) Hydrogen bond between CASP3 and naringenin. (b) Hydrogen bonds between STAT3 and cryptotanshinone.

The lower the binding energy, the stronger the binding force between the protein and the drug molecule. Affinity < −4.25 kcal/mol indicates there is possibility of binding between the ligand and the receptor, affinity < −5.00 kcal/mol indicates good binding strength, and affinity < −7.00 kcal/mol indicates satisfactory binding strength [24]. In the molecular docking results mentioned above, only two groups showed good affinity (<−5.00 kcal/mol). Therefore, through the auxiliary verification of molecular docking, we obtained two relatively valuable protein targets (CASP and STAT3) for experimental verification in the future. The two groups, with good docking results, were further processed to obtain a visualized molecular docking simulation map (Figures 5(a) and 5(b)).

Figure 5(a) presents one hydrogen bond between CASP3 and naringenin, formed by the hydrogen atom (H) of diosgenin and the oxygen atom (OD1) of the 275th amino acid on the B chain of CASP3, Phenylalanine (PHE257). The result of STAT3 docking with cryptotanshinone, Figure 5(b), showed two hydrogen bonds. One hydrogen bond is formed by the oxygen atom (O) of cryptotanshinone and the hydrogen atom (HN) of the 386th amino acid on the A chain of STAT3, Isoleucine (ILE386). Another hydrogen bond is formed by another oxygen atom (O) of cryptotanshinone and the hydrogen atom (H2D2) of the 390th amino acid on the A chain of STAT3, Asparagine (ASN390).

### 3.3. Signal Pathway Enrichment Analysis

**Figure 6:**
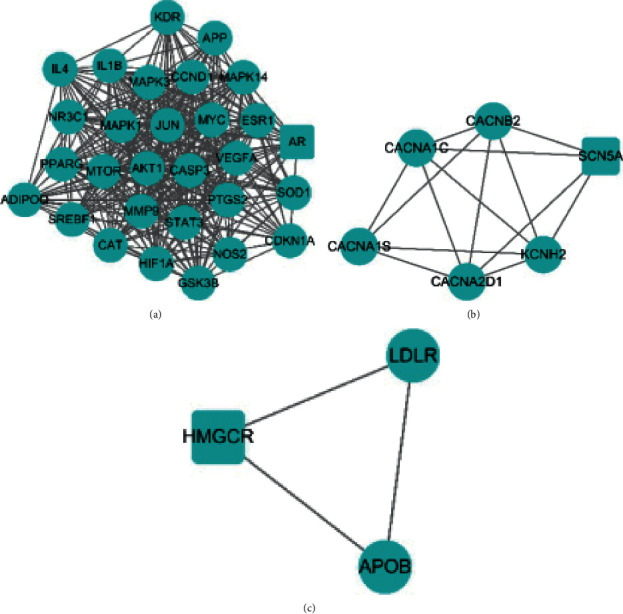
Network diagram of three gene clusters. (a) Cluster 1. (b) Cluster 2. (c) Cluster 3.

With the assistance of MCODE analysis, three candidate gene clusters of the constructed PPI network were obtained (Figure 6). Meanwhile, details of clusters are presented in Table 5.

Two modules with higher score and larger number of genes were selected for follow-up research. The gene information of gene cluster 1 and cluster 2 were uploaded to the DAVID database, and signal pathways such as the HIF-1 signaling pathway, TNF signaling pathway, and VEGF signaling pathway were obtained (Tables 6(a) and 6(b)). These signal pathways are the key to study the potential mechanism of Qiweitangping in treating diabetic CHD.

KEGG pathway enrichment analysis results show that Qiweitangping in the treatment of diabetic CHD involves the HIF-1 signaling pathway, PI3K-Akt signaling pathway, ErbB signaling pathway, and other signaling pathways.

The HIF-1 signaling pathway can promote cardiomyocytes to adapt to ischemia and hypoxia by affecting VEGF expression, mitochondrial function, cell apoptosis, antioxidative stress, etc., to protect or restore their functions [25]. HIF-1*α* regulates the expression level of downstream functional genes, mediating the response of organs and tissues to microcirculation hypoxia, and participates in the occurrence and development of diabetes complications [26]. Knocking out HIF-1 gene aggravates pressure-overload-induced myocardial fibrosis, cardiomyocyte hypertrophy, decreased myocardial capillary density and myocardial cell apoptosis, and promotes heart failure [27].

The JAK/STAT3 signaling pathway is a modulator of cytokine signal transduction, which can be used in a variety of stress responses, including ischemia, hypoxia, and oxidative stress, and plays an important role in mediating insulin resistance damage to myocardial protection [21]. Animal experiments have found that inhibiting PI3K/AKT signal activity can protect atherosclerotic mice [28].

The ErbB signaling pathway plays an important role in the maintenance of cardiac function. In cardiac tissue, neuregulin-1 can bind to ErbB4 receptors and form heterodimers with ErbB2 receptors. Phosphorylation of specific tyrosine residues on the protein takes place, thereby activating the PI3K/Akt signaling pathway [29].

The U.S. Food and Drug Administration issued an announcement stating that GnRH agonist drugs lead to an increased incidence of cardiovascular disease and diabetes. This indicates that the GnRH signaling pathway may be related to the occurrence of diabetic CHD [30].

### 3.4. Visualizing Multivariate Network

**Figure 7:**
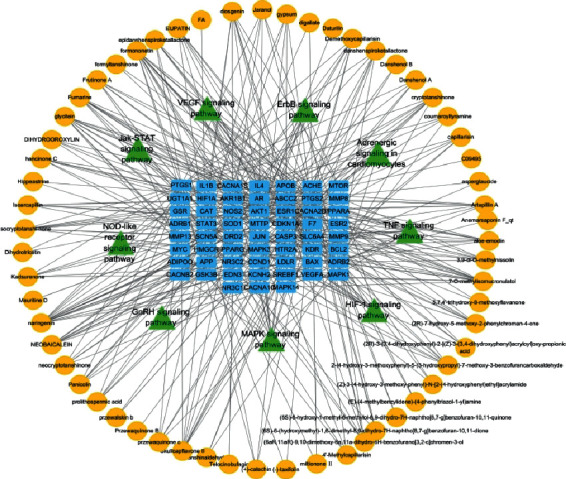
Visualized multivariate network.

To clarify the relationship between drug chemical components, candidate genes, and signaling pathways, a visualized multivariate network, basing on the data collected in the database, was constructed (Figure 7).

Fifty-nine blue square nodes represent target genes, sixty-two yellow nodes represent chemical components, and nine green triangles represent signaling pathways.

The “drug component-gene target-pathway” network displays the associations between drugs, candidate gene targets, and main signal pathways with lines. The density of the lines in the network reflects the complexity of the mechanism of action and the degree of association between the elements. This network visualizes the treatment mechanism of multicomponent, multitarget, and multisignal pathways. It is consistent with Liu's “harmonious whole” medical theory.

## 4. Conclusions

This study lays the foundation for further research on the mechanism of Qiweitangping's disease treatment. Subsequent researchers may use the genes and signal pathways obtained in this study to conduct further experimental verification to determine the mechanism, for example, detecting changes in gene expression of the candidate genes we obtained such as AKT1, CASP3, VEGF-A, testing changes in signal pathways such as the HIF-1 signaling pathway, PI3K-Akt signaling pathway, and ErbB signaling pathway, and studying biological processes such as apoptosis, angiogenesis, and inflammation. Meanwhile, the verification results of the molecular docking of STAT3 and CASP are good, which means better expectations for the experimental study of the expression of these two genes.

Although this article has found some potential targets and pathways of Qiweitangping in the treatment of diabetic CHD through network pharmacology, what we know is still not enough to clarify all the actual mechanisms of action of the drug.

Compared with Western medicine, Liu's medical theory, like traditional Chinese medical theories, treats the whole human body and treats diseases from a macroperspective. When treating diabetes, a complex disease that can cause multiple complications, Qiweitangping, developed based on the thinking of “coordinating the whole, highlighting the local,” performs well in clinical practice. Compared with Western medicine, Chinese herbal medicine not only can treat diseases in all directions and multiple targets but also has milder medicinal properties and will not cause obvious side effects to the human body while treating.

However, Qiweitangping's shortcoming is also obvious. Patients often need to take drugs for months or even years to improve their condition. Therefore, patients often need to use it together with common drugs in the early stage of treatment.

## Data Availability

The data used to support the study are available from the corresponding author (e-mail: tliu15@qub.ac.uk).

## Abbreviations

AKT1: AKT serine/threonine kinase 1
CASP3: Caspase 3
CHD: Coronary heart disease
DL: Drug-likeness
ErbB: Receptor tyrosine kinase
GnRH: Gonadotropin-releasing hormone
HIF-1a: Hypoxia-inducible factor 1 subunit alpha
KEGG: Kyoto Encyclopedia of Genes and Genomes
MAPK1: Mitogen-activated protein kinase 1
MAPK3: Mitogen-activated protein kinase 3
OB: Oral bioavailability
STAT3: Signal transducer and activator of transcription 3
VEGF-A: Vascular endothelial growth factor A.

## Supplementary Materials

Supplementary Materials Table 1: the chemical components of Qiweitangping. Table 2: candidate genes in the treatment. Table 3: PPI network graph data statistics. Table 4: molecular docking binding energy. Table 5: MCODE cluster analysis detailed information table. Table 6: potential signal pathways of Qiweitangping in the treatment of diabetic CHD.
