# Supplementary material for: Network Pharmacological Study and Molecular Docking Analysis of Qiweitangping in Treating Diabetic Coronary Heart Disease
Source: Evid Based Complement Alternat Med. 2021 Jul 27;2021:9925556. doi: 10.1155/2021/9925556 (PMC8337130; doi:10.1155/2021/9925556)
Supplement: Supplementary Materials — Table 1: the chemical components of Qiweitangping. Table 2: candidate genes in the treatment. Table 3: PPI network graph data statistics. Table 4: molecular docking binding energy. Table 5: MCODE cluster analysis detailed information table. Table 6: potential signal pathways of Qiweitangping in the treatment of diabetic CHD. [file 9925556.f1.zip › 9925556.f1/Supplementary Description.docx]

**Supplementary Description:**

Table 1: The chemical components of Qiweitangping. Table 2: Candidate genes in the treatment. Table 3: PPI network graph data statistics. Table 4: Molecular docking binding energy. Table 5: MCODE cluster analysis detailed information table. Table 6: Potential signal pathways of Qiweitangping in the treatment of diabetic CHD
