# Supplementary material for: Network Pharmacological Study and Molecular Docking Analysis of Qiweitangping in Treating Diabetic Coronary Heart Disease
Source: Evid Based Complement Alternat Med. 2021 Jul 27;2021:9925556. doi: 10.1155/2021/9925556 (PMC8337130; doi:10.1155/2021/9925556)
Supplement: Supplementary Materials — Table 1: the chemical components of Qiweitangping. Table 2: candidate genes in the treatment. Table 3: PPI network graph data statistics. Table 4: molecular docking binding energy. Table 5: MCODE cluster analysis detailed information table. Table 6: potential signal pathways of Qiweitangping in the treatment of diabetic CHD. [file 9925556.f1.zip › 9925556.f1/Supplementary file 1. The chemical components of Qiweitangping.docx]

Table 1: The chemical components of Qiweitangping

| Mol ID | Molecule Name | OB (%) | DL |
| --- | --- | --- | --- |
| MOL001736 | (-)-taxifolin | 60.51 | 0.27 |
| MOL000492 | (+)-catechin | 54.83 | 0.24 |
| MOL007132 | (2R)-3-(3,4-dihydroxyphenyl)-2-[(Z)-3-(3,4-dihydroxyphenyl) acryloyl]oxy-propionic acid | 109.38 | 0.35 |
| MOL000228 | (2R)-7-hydroxy-5-methoxy-2-phenylchroman-4-one | 55.23 | 0.2 |
| MOL000380 | (6aR,11aR)-9,10-dimethoxy-6a,11a-dihydro-6H-benzofurano[3,2-c] chromen-3-ol | 64.26 | 0.42 |
| MOL007155 | (6S)-6-(hydroxymethyl)-1,6-dimethyl-8,9-dihydro-7H-naphtho[8,7-g] benzofuran-10,11-dione | 65.26 | 0.45 |
| MOL007150 | (6S)-6-hydroxy-1-methyl-6-methylol-8,9-dihydro-7H-naphtho[8,7-g] benzofuran-10,11-quinone | 75.39 | 0.46 |
| MOL012893 | (E)-(4-methylbenzylidene) -(4-phenyltriazol-1-yl)amine | 57.87 | 0.19 |
| MOL000483 | (Z)-3-(4-hydroxy-3-methoxy-phenyl)-N-[2-(4-hydroxyphenyl) ethyl]acrylamide | 118.35 | 0.26 |
| MOL007050 | 2-(4-hydroxy-3-methoxyphenyl)-5-(3-hydroxypropyl)-7-methoxy-3-benzofurancarboxaldehyde | 62.78 | 0.4 |
| MOL000371 | 3,9-di-O-methylnissolin | 53.74 | 0.48 |
| MOL008045 | 4'-Methylcapillarisin | 72.18 | 0.35 |
| MOL012246 | 5,7,4'-trihydroxy-8-methoxyflavanone | 74.24 | 0.26 |
| MOL000378 | 7-O-methylisomucronulatol | 74.69 | 0.3 |
| MOL000471 | aloe-emodin | 83.38 | 0.24 |
| MOL004489 | Anemarsaponin F_qt | 60.06 | 0.79 |
| MOL008047 | Artepillin A | 68.32 | 0.24 |
| MOL001677 | asperglaucide | 58.02 | 0.52 |
| MOL010913 | C09495 | 77.09 | 0.25 |
| MOL008043 | capillarisin | 57.56 | 0.31 |
| MOL000631 | coumaroyltyramine | 112.9 | 0.2 |
| MOL007088 | cryptotanshinone | 52.34 | 0.4 |
| MOL007082 | Danshenol A | 56.97 | 0.52 |
| MOL007081 | Danshenol B | 57.95 | 0.56 |
| MOL007094 | danshenspiroketallactone | 50.43 | 0.31 |
| MOL008397 | Daturilin | 50.37 | 0.77 |
| MOL008046 | Demethoxycapillarisin | 52.33 | 0.25 |
| MOL000569 | digallate | 61.85 | 0.26 |
| MOL002937 | DIHYDROOROXYLIN | 66.06 | 0.23 |
| MOL007813 | Dihydrotricetin | 58.12 | 0.28 |
| MOL000546 | diosgenin | 80.88 | 0.81 |
| MOL007105 | epidanshenspiroketallactone | 68.27 | 0.31 |
| MOL002235 | EUPATIN | 50.8 | 0.41 |
| MOL000433 | FA | 68.96 | 0.71 |
| MOL000392 | formononetin | 69.67 | 0.21 |
| MOL007058 | formyltanshinone | 73.44 | 0.42 |
| MOL005321 | Frutinone A | 65.9 | 0.34 |
| MOL000787 | Fumarine | 59.26 | 0.83 |
| MOL008400 | glycitein | 50.48 | 0.24 |
| MOL005430 | hancinone C | 59.05 | 0.39 |
| MOL004497 | Hippeastrine | 51.65 | 0.62 |
| MOL008039 | Isoarcapillin | 57.4 | 0.41 |
| MOL007108 | isocryptotanshi-none | 54.98 | 0.39 |
| MOL000354 | isorhamnetin | 49.6 | 0.31 |
| MOL000239 | Jaranol | 50.83 | 0.29 |
| MOL000322 | Kadsurenone | 54.72 | 0.38 |
| MOL013271 | Kokusaginin | 66.68 | 0.2 |
| MOL000211 | Mairin | 55.38 | 0.78 |
| MOL012992 | Mauritine D | 89.13 | 0.45 |
| MOL007120 | miltionone Ⅱ | 71.03 | 0.44 |
| MOL004328 | naringenin | 59.29 | 0.21 |
| MOL002934 | NEOBAICALEIN | 104.34 | 0.44 |
| MOL007125 | neocryptotanshinone | 52.49 | 0.32 |
| MOL007835 | orobanchoside_qt | 55.99 | 0.82 |
| MOL002932 | Panicolin | 76.26 | 0.29 |
| MOL007130 | prolithospermic acid | 64.37 | 0.31 |
| MOL007064 | przewalskin b | 110.32 | 0.44 |
| MOL007068 | Przewaquinone B | 62.24 | 0.41 |
| MOL007069 | przewaquinone c | 55.74 | 0.4 |
| MOL002927 | Skullcapflavone II | 69.51 | 0.44 |
| MOL007079 | tanshinaldehyde | 52.47 | 0.45 |
| MOL005531 | Telocinobufagin | 69.99 | 0.79 |
