# Supplementary material for: Network Pharmacological Study and Molecular Docking Analysis of Qiweitangping in Treating Diabetic Coronary Heart Disease
Source: Evid Based Complement Alternat Med. 2021 Jul 27;2021:9925556. doi: 10.1155/2021/9925556 (PMC8337130; doi:10.1155/2021/9925556)
Supplement: Supplementary Materials — Table 1: the chemical components of Qiweitangping. Table 2: candidate genes in the treatment. Table 3: PPI network graph data statistics. Table 4: molecular docking binding energy. Table 5: MCODE cluster analysis detailed information table. Table 6: potential signal pathways of Qiweitangping in the treatment of diabetic CHD. [file 9925556.f1.zip › 9925556.f1/Supplementary file 2. Candidate genes in the treatment.docx]

Table 2: Candidate genes in the treatment

| Candidate genes | | | | | |
| --- | --- | --- | --- | --- | --- |
| PTGS1 | BAX | PPARG | DRD2 | HIF1A | HMGCR |
| PTGS2 | CASP3 | MAPK14 | MMP13 | ABCC2 | UGT1A1 |
| NOS2 | MYC | GSK3B | MMP8 | MTOR | PPARA |
| AR | IL1B | NR3C2 | JUN | MMP9 | SREBF1 |
| F7 | ESR1 | NR3C1 | IL4 | BCL2 | GSR |
| ESR2 | CAT | ACHE | ADRB1 | MAPK3 | ADIPOQ |
| SCN5A | KCNH2 | STAT3 | CACNA2D1 | MAPK1 | CACNA1C |
| KDR | ADRB2 | CCND1 | AKT1 | LDLR | CACNB2 |
| AKR1B1 | SLC6A4 | APP | VEGFA | MTTP | HTR2A |
| CDKN1A | CACNA1S | EDN3 | SOD1 | APOB |  |
