# Supplementary material for: Network Pharmacological Study and Molecular Docking Analysis of Qiweitangping in Treating Diabetic Coronary Heart Disease
Source: Evid Based Complement Alternat Med. 2021 Jul 27;2021:9925556. doi: 10.1155/2021/9925556 (PMC8337130; doi:10.1155/2021/9925556)
Supplement: Supplementary Materials — Table 1: the chemical components of Qiweitangping. Table 2: candidate genes in the treatment. Table 3: PPI network graph data statistics. Table 4: molecular docking binding energy. Table 5: MCODE cluster analysis detailed information table. Table 6: potential signal pathways of Qiweitangping in the treatment of diabetic CHD. [file 9925556.f1.zip › 9925556.f1/Supplementary file 4. Molecular docking binding energy.docx]

Table 4: Molecular docking binding energy

| Target | compound | binding energy/(kcal·mol−1) |
| --- | --- | --- |
| AKT1 | naringenin | -2.77 |
| AKT1 | diosgenin | -2.65 |
| CASP3 | naringenin | -5.89 |
| VEGF-A | diosgenin | -3.6 |
| STAT3 | cryptotanshinone | -5.36 |
