# Supplementary material for: Network Pharmacological Study and Molecular Docking Analysis of Qiweitangping in Treating Diabetic Coronary Heart Disease
Source: Evid Based Complement Alternat Med. 2021 Jul 27;2021:9925556. doi: 10.1155/2021/9925556 (PMC8337130; doi:10.1155/2021/9925556)
Supplement: Supplementary Materials — Table 1: the chemical components of Qiweitangping. Table 2: candidate genes in the treatment. Table 3: PPI network graph data statistics. Table 4: molecular docking binding energy. Table 5: MCODE cluster analysis detailed information table. Table 6: potential signal pathways of Qiweitangping in the treatment of diabetic CHD. [file 9925556.f1.zip › 9925556.f1/Supplementary file 5. MCODE cluster analysis detailed information table.docx]

Table 5: MCODE cluster analysis detailed information table

| Cluster | Score | Nodes | Edges | Genes |
| --- | --- | --- | --- | --- |
| 1 | 23.929 | 29 | 335 | MAPK1, JUN, CAT, SOD1, MYC, HIF1A, PTGS2, SREBF1, CCND1, CDKN1A, NR3C1, GSK3B, KDR, AR, VEGFA, MTOR, AKT1, APP, MMP9, CASP3, NOS2, IL4, IL1B, ESR1, PPARG, ADIPOQ, STAT3, MAPK14, MAPK3 |
| 2 | 5.6 | 6 | 14 | CACNA1S, CACNB2, KCNH2, SCN5A, CACNA2D1, CACNA1C |
| 3 | 3 | 3 | 3 | HMGCR, APOB, LDLR |
