# Supplementary material for: Network Pharmacological Study and Molecular Docking Analysis of Qiweitangping in Treating Diabetic Coronary Heart Disease
Source: Evid Based Complement Alternat Med. 2021 Jul 27;2021:9925556. doi: 10.1155/2021/9925556 (PMC8337130; doi:10.1155/2021/9925556)
Supplement: Supplementary Materials — Table 1: the chemical components of Qiweitangping. Table 2: candidate genes in the treatment. Table 3: PPI network graph data statistics. Table 4: molecular docking binding energy. Table 5: MCODE cluster analysis detailed information table. Table 6: potential signal pathways of Qiweitangping in the treatment of diabetic CHD. [file 9925556.f1.zip › 9925556.f1/Supplementary file 6. Potential signal pathways of Qiweitangping in the treatment of diabetic CHD.docx]

Table 6: Potential signal pathways of Qiweitangping in the treatment of diabetic CHD

Table 6a. Pathways of cluster 1

| Term | Count | PValue | Genes |
| --- | --- | --- | --- |
| HIF-1 signaling pathway | 9 | 4.60E-09 | AKT1, MAPK1, CDKN1A, HIF1A, MAPK3, VEGFA, MTOR, NOS2, STAT3 |
| TNF signaling pathway | 9 | 6.36E-09 | AKT1, MAPK1, CASP3, PTGS2, MAPK14, JUN, MMP9, MAPK3, IL1B |
| Prolactin signaling pathway | 8 | 1.25E-08 | AKT1, MAPK1, CCND1, GSK3B, MAPK14, MAPK3, ESR1, STAT3 |
| Thyroid hormone signaling pathway | 9 | 1.45E-08 | AKT1, MAPK1, CCND1, HIF1A, GSK3B, MAPK3, ESR1, MTOR, MYC |
| ErbB signaling pathway | 8 | 5.34E-08 | AKT1, MAPK1, CDKN1A, GSK3B, JUN, MAPK3, MTOR, MYC |
| VEGF signaling pathway | 7 | 1.41E-07 | AKT1, MAPK1, PTGS2, MAPK14, MAPK3, VEGFA, KDR |
| PI3K-Akt signaling pathway | 11 | 8.36E-07 | AKT1, IL4, MAPK1, CCND1, CDKN1A, GSK3B, MAPK3, VEGFA, MTOR, MYC, KDR |
| FoxO signaling pathway | 8 | 9.74E-07 | AKT1, MAPK1, CCND1, CDKN1A, MAPK14, MAPK3, CAT, STAT3 |
| AMPK signaling pathway | 7 | 1.21E-05 | SREBF1, AKT1, CCND1, PPARG, MTOR, ADIPOQ |
| Estrogen signaling pathway | 6 | 4.32E-05 | AKT1, MAPK1, JUN, MMP9, MAPK3, ESR1 |
| MAPK signaling pathway | 8 | 5.59E-05 | AKT1, MAPK1, CASP3, MAPK14, JUN, MAPK3, IL1B, MYC |
| Fc epsilon RI signaling pathway | 5 | 1.56E-04 | AKT1, IL4, MAPK1, MAPK14, MAPK3 |
| Insulin signaling pathway | 6 | 2.33E-04 | SREBF1, AKT1, MAPK1, GSK3B, MAPK3, MTOR |
| Oxytocin signaling pathway | 6 | 3.15E-04 | MAPK1, CCND1, CDKN1A, PTGS2, JUN, MAPK3 |
| Serotonergic synapse | 5 | 8.77E-04 | MAPK1, APP, CASP3, PTGS2, MAPK3 |
| NOD-like receptor signaling pathway | 4 | 0.001225023 | MAPK1, MAPK14, MAPK3, IL1B |
| Rap1 signaling pathway | 6 | 0.001692646 | AKT1, MAPK1, MAPK14, MAPK3, VEGFA, KDR |
| mTOR signaling pathway | 4 | 0.001884513 | AKT1, MAPK1, MAPK3, MTOR |
| Jak-STAT signaling pathway | 5 | 0.004369671 | AKT1, IL4, CCND1, MYC, STAT3 |
| GnRH signaling pathway | 4 | 0.005047236 | MAPK1, MAPK14, JUN, MAPK3 |
| Chemokine signaling pathway | 5 | 0.005549811 | AKT1, MAPK1, GSK3B, MAPK3, STAT3 |
| Ras signaling pathway | 5 | 0.013604293 | AKT1, MAPK1, MAPK3, VEGFA, KDR |
| Sphingolipid signaling pathway | 4 | 0.014672412 | AKT1, MAPK1, MAPK14, MAPK3 |
| Platelet activation | 4 | 0.015330173 | AKT1, MAPK1, MAPK14, MAPK3 |
| Wnt signaling pathway | 4 | 0.018127648 | CCND1, GSK3B, JUN, MYC |
| Adrenergic signaling in cardiomyocytes | 4 | 0.019245746 | AKT1, MAPK1, MAPK14, MAPK3 |
| p53 signaling pathway | 3 | 0.032344437 | CCND1, CDKN1A, CASP3 |
| PPAR signaling pathway | 3 | 0.0350969 | PPARG, ADIPOQ |
| TGF-beta signaling pathway | 3 | 0.044896505 | MAPK1, MAPK3, MYC |
| cAMP signaling pathway | 4 | 0.049552741 | AKT1, MAPK1, JUN, MAPK3 |
| Cholinergic synapse | 3 | 0.076799767 | AKT1, MAPK1, MAPK3 |
| Dopaminergic synapse | 3 | 0.096131336 | AKT1, GSK3B, MAPK14 |

Table 6b. Pathways of cluster 2

| Term | Count | PValue | Genes |
| --- | --- | --- | --- |
| Adrenergic signaling in cardiomyocytes | 5 | 1.45E-07 | CACNA2D1, CACNB2, CACNA1C, CACNA1S, SCN5A |
| Oxytocin signaling pathway | 4 | 4.10E-05 | CACNA2D1, CACNB2, CACNA1C, CACNA1S |
| MAPK signaling pathway | 4 | 1.82E-04 | CACNA2D1, CACNB2, CACNA1C, CACNA1S |
| Renin secretion | 2 | 0.037756611 | CACNA1C, CACNA1S |
| Aldosterone synthesis and secretion | 2 | 0.045293351 | CACNA1C, CACNA1S |
| Insulin secretion | 2 | 0.048756874 | CACNA1C, CACNA1S |
| GnRH signaling pathway | 2 | 0.049909285 | CACNA1C, CACNA1S |
| GABAergic synapse | 2 | 0.051060648 | CACNA1C, CACNA1S |
| Cholinergic synapse | 2 | 0.064226249 | CACNA1C, CACNA1S |
| Vascular smooth muscle contraction | 2 | 0.067638106 | CACNA1C, CACNA1S |
| cGMP-PKG signaling pathway | 2 | 0.092374151 | CACNA1C, CACNA1S |
